# Literature Review of Proteomics Approach Associated with Coffee

**DOI:** 10.3390/foods13111670

**Published:** 2024-05-27

**Authors:** Shah Zaman, Zhiguo Shan

**Affiliations:** School of Tea & Coffee, Pu’er University, Pu’er 665000, China; shahzamantea@163.com

**Keywords:** *Coffea* *arabica*, environment adaptation, proteomics approach, somatic embryogenesis

## Abstract

As a significant crop growing all across the world, coffee is mostly produced in the bean belt of our global atlas. Worldwide variations in environmental conditions are causing a decline in the yield and quality of coffee varieties. Coffee production is the main emphasis of several traditional breeding techniques. But conventional breeding methods are not sufficient to tackle the problems related to coffee. The field of genomics, which includes transcriptomics, proteomics, and metabolomics, has made great paces in the last ten years. Proteomics is a well-known technique used to enhance the growth, yield, breeding, and quality of different plants under stable and shifting environments. The regulation of specific enzymes, genes, protein expression, modification, translation, and other features played an important role in the enhancement of important plants. However, relatively less research on the proteomics approach for coffee has been published in the last few years. For this reason, some of the most important aspects of proteome profiling for coffee plants have been covered in this review, including growth, the somatic embryo technique, altitude, environmental adoption, drought, and the role that proteins and important enzymes play in the flavor and taste of coffee. This review can aid in the breeding of new cultivars and improve coffee attributes. Furthermore, the present literature can pave the way for proteomics research on coffee.

## 1. Introduction

**Figure 1:**
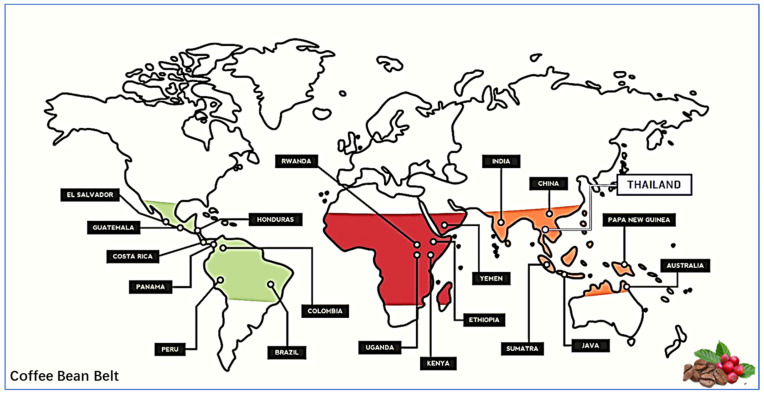
The “Coffee Bean Belt” on the world atlas. This figure was adapted from [22] with some modifications. The coffee-growing countries are mentioned with names in each continent. Green color indicates the coffee growing areas in the South American continent; maroon color indicates the coffee production areas in the African continent and Middle Eastern region of the Asian continent; orange color represents coffee growing areas in Asia and Oceania.

Coffee ranks among the top three beverages globally [1] and is the second most consumed drink [2]. In fact, coffee is acknowledged as one of the most actively traded commodities on a global scale [3], and it has a substantial economic influence on the countries that produce it. Drinking of coffee has increased significantly over the past decade [4]. *Coffea arabica* was the inaugural species of the Coffea genus to be commercially farmed, thus establishing itself as the benchmark for beverage excellence. Despite the existence of 130 distinct species within the Coffea genus, only two of them, namely *Coffea arabica* (Arabica coffee) and *Coffea canephora* (Robusta coffee), are currently subject to commercial exploitation. *Coffea arabica* accounts for 61% of global production [5]. Worldwide coffee production, encompassing both arabica and robusta varieties, was projected to have amounted to an estimated 10.368 billion tons in 2022/23 [6]. Brazil holds the distinction of being the foremost global producer and exporter of freshly harvested coffee [7]. Coffee plants grown in the Amazonian region are assumed to be the most premium coffee [8]. The quality of coffee beverages is determined by a delicate equilibrium between several circumstances, actions, and decisions, which begin in the field during cultivation and extend to the storage, processing, and roasting of the beans. Both non-genetic and genetic factors have been demonstrated to impact the quality of beverages. The quality of coffee beverages can be influenced by various factors, including soil fertility, fertilizer, climate, altitude, plant health, the stage of fruit maturation at harvest, post-harvest processing, and genetic background. In fact, coffee is not grown everywhere in the world due to varying environmental conditions and geographical locations. The optimal conditions for coffee tree growth are observed globally within the equatorial zone, also known as “The Bean Belt”. As shown in Figure 1, this region spans from latitudes 25 degrees north to 30 degrees south. Fortunately, the Yunnan province in southwestern China is located within the coffee belt and is responsible for 80% of coffee production, with Hainan accounting for the remaining 20%. The coffee cultivation regions include the cities of Pu’er, Baoshan, and Xishuangbanna, which are situated near Southeast Asian nations such as Laos, Cambodia, and Vietnam. Pu’er city has emerged as a major coffee producer in recent years, contributing to 95% of China’s coffee production. This is primarily due to the ideal climatic conditions in the region. Pu’er coffee is grown at an altitude of approximately 1000 m, resulting in a low level of acidity. As a result, China now ranks as the 13th largest coffee producer globally. Over the past thirty years, the coffee industry’s status among the eight mainstream typical industries in Yunnan Province was solidified with a total production yield of 109,100 tons [9,10].

The excellent coffee genome-wide sequence has made it feasible to identify the gene and protein components in coffee cherries by using transcriptomic and proteomic analytical approaches [11]. Transcriptomics by itself was unable to precisely forecast the amount of proteins and the activity of proteases [12]. Low-molecular-weight metabolites and high-molecular-weight proteins as well as the dynamic state of the cell could be analyzed qualitatively and quantitatively using combined metabolomics and proteomics [13]. Amino acids, peptides, and proteins are widely recognized for their significant contribution to the production of coffee aroma [14]. The literature reports a wide range of protein concentrations in *Coffea arabica* and *Coffea canephora* beans, ranging from 10 to 15%. However, this difference is due to the different methodologies used to evaluate proteins [15,16]. Approximately 33% of the total protein found in coffee beans is believed to attach to arabinogalactans in the cell wall. However, there is a lack of specific knowledge about the characteristics of these proteins [17].

Most of the proteins are likely to be enzymes, such as polyphenol oxidase and peroxidases, with the majority of them located in the cytoplasm [16]. The concentration of free amino acids in *Coffea arabica* and *Coffea canephora* types is significantly lower than that of proteins, ranging from 0.15% to 2.5%. Despite this fact, these chemicals represent the most significant group of substances associated with the formation of coffee flavor. Among the types of *C. canephora*, the amounts of all free amino acids are higher compared to *C. arabica,* except for glutamate [17]. In fact, the importance of the different reactions in the determination of food quality, including the quality of coffee, is comprehended; however, proteins have been neglected in genetic studies aiming to select/breed coffee for quality.

In studies published by the United States Department of Agriculture, 2022 coffee beans obtained from *C. arabica* were widely acknowledged to possess a more robust, flavorful, and harmonious flavor profile in comparison to the bitter *C. canephora* [18,19]. Consequently, these beans constitute approximately 55–70% of global coffee production. Regrettably, it has been observed that *C. arabica* plants have a heightened susceptibility to both disease and climatic conditions. Consequently, it is imperative to conduct additional research to explore the influence of these factors on *C. arabica* in order to enhance coffee output [20], including the use of modern technologies such as transcriptomics, proteomics, and genomics. Due to coffee’s spread in different continents and complex genetic background, it is hard to understand the coffee mechanism through classic genetic breeding, which is also time-consuming [21]. So, the utilization of the quick and smart strategy of omics technology including proteomics analysis on coffee attributes might be a positive step for enhancing the growth, development, and breeding of coffee.

## 2. Integration of Proteomic Technology

The advancement of omics has facilitated the comprehensive investigation of several biological processes at the molecular level across the entire genome. The genomic and proteomic levels have been extensively examined in the realm of plant biology, specifically in relation to growth and development, organogenesis, stress response, and fundamental processes such as organelle biogenesis, cell cycle, and metabolism [23]. The field of proteomics is of paramount importance in the investigation of cellular differentiation, signal transmission, cancer progression, and therapeutic interventions. In recent years, there has been a growing significance in the field of plant protein research. The regulation of plant proteomes encompasses a wide range of functional, geographical, and temporal variability. This diversity is managed by several variables that consistently influence protein abundance, modifications, interactions, localization, and activity to adapt to the ever-changing requirements of plants. The identification of proteins involved in crucial physiological processes, such as photosynthesis, carbon assimilation, and nutritional intake, can be achieved through the utilization of proteomic analysis by researchers. Through comprehending the proteomic foundation of high-yielding cultivars, breeders can strategically choose or genetically modify crops to enhance the efficiency of energy capture and usage, hence augmenting productivity, and proteomics has demonstrated its worth in enhancing the resistance of crops to abiotic stresses, parasites, and diseases. Through the comparison of proteomes between susceptible and resistant varieties, scientists are able to discern proteins that are linked to stress tolerance, defense mechanisms, or signaling pathways. By utilizing this understanding, it is possible to create cultivars that are more resistant to biotic and abiotic stresses via genetic engineering or conventional breeding approaches [24,25,26].

This review focuses on specific proteome results in coffee and offers an update on recent advancements and the prospect of rapidly developing proteomic methods for enhancing crop quality.

## 3. Aim of the Current Article

**Figure 2:**
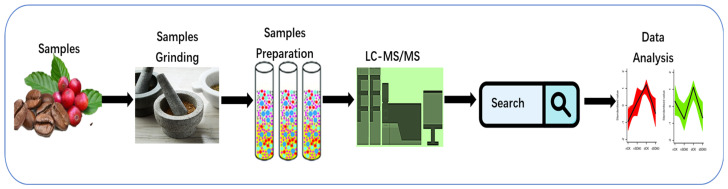
The proteomics approach associated with coffee.

A preliminary search was conducted on PubMed using the keywords “Proteomics” and “Coffee” with the publication year range of 2014 to 2024 selected. Surprisingly, only 10 papers were found as a result. After a thorough assessment, irrelevant articles were excluded, leaving only five relevant papers for the current review. This paper has five main objectives: 1. highlighting the significance of the proteomics approach in studying the flavor and growth stages of coffee; 2. presenting the findings on the proteomics approach’s impact on coffee altitudes and coffee’s ability to adapt to different environmental conditions; 3. understanding the effects of drought on coffee through proteomic studies; 4. exploring the connection between proteomics and the stages of somatic embryogenesis in coffee; 5. examining existing studies, identifying gaps, and proposing future directions for the proteomics approach in coffee research (Figure 2).

## 4. Proteomics Approach Associated with Flavor of Arabica Coffee

The impact of environmental conditions on flavor is undeniably significant; it is important to acknowledge that proteomic variations among cultivars, which arise from extended adaptation to specific climatic niches, are also expected to have a significant influence on various metabolic processes that contribute to specific aspects of coffee plant and bean quality. These factors encompass disease resistance, maintenance of homeostasis in response to environmental conditions, and potentially the production or breakdown of flavor precursors. Although the coffee industry plays a significant role in the worldwide economy, there have been limited proteomic studies that compare different coffee cultivars or even different species of coffee [20]. In recent years, a number of studies have repeatedly validated the significance of the protein and/or amino acid composition and profile in coffee beans, highlighting their crucial role in determining the quality of coffee [14,27,28], several amino acids and proteins associated with coffee dispatched in Table 1.

**Figure 3:**
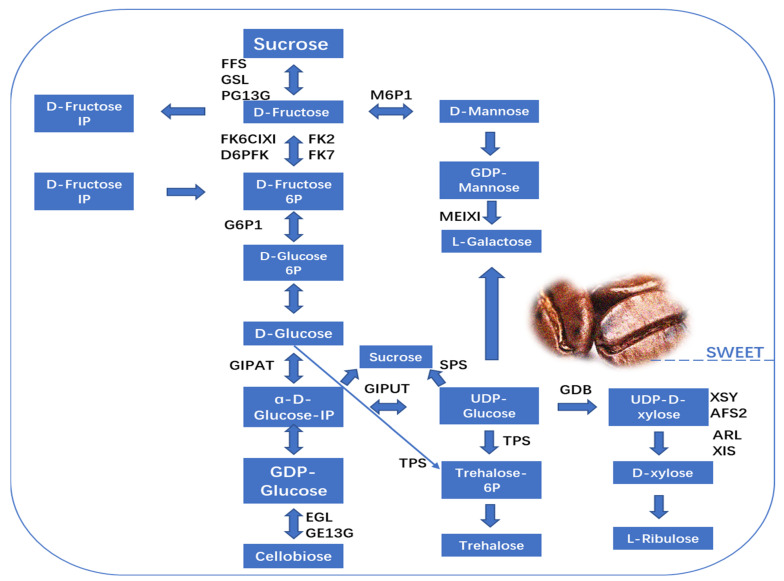
Different enzymes that participate in the metabolic pathways of sugar, contributing to the sweetness of coffee. This figure was adapted from [32] with some modifications.

Recently, a TMT labeling proteomics study was performed to identify the molecular mechanism behind variations in nonvolatile chemicals in coffee cherries at different maturation stages, and the author found 11,309 peptides; the length of the peptides mainly ranged from 14 to 26, and the molecular weights of proteins ranged from 10 to 100 kDa, suggesting the appropriate molecular weight. In fact, the key proteins were beta-galactosidase, cysteine protease, aminomethyltransferase, carbohydrate synthetic protease, amino acid synthetic protease, lipase, and enzymes related to the synthesis of flavonoid polyphenols. Furthermore, Gene Ontology analysis revealed that the proteins participated in molecular, biological, and metabolic processes, and the enriched KEGG pathways in four different stages of coffee were amino sugar, glutathione metabolism, fructose and mannose metabolism, nucleotide sugar metabolism, flavonoid biosynthesis, inositol phosphate metabolism, and fatty acid degradation [32]. Due to the above activities and the regulation of enzymes, the majority of metabolites have been demonstrated to directly contribute to flavor or act as precursors to flavor [36]. Flavonoids have the potential to impart a bitter and astringent flavor profile, while free amino acids are known to contribute to an umami taste. Sugar, on the other hand, plays a role in imparting a sweet taste, and organic acids contribute to the development of a sour taste [37]. Therefore, the subsequent examinations mostly concentrated on the biosynthesis and metabolism protocols of organic acids, amino acids, flavonoids, and sugars, as well as the impact of pertinent proteases during the four phases of coffee cherry maturation [32]. Interestingly, the levels of β-fructofuranosidase and α-glucosidase are positively regulated with sucrose and fructose. This suggests that the presence of these enzymes contributes to the buildup of the two reducing sugars throughout the process of ripening, and glucan 1,3-α-glucosidase and glucan 1,3-α-glucosidase resulted in a negative association with the proliferation of sucrose and fructose. In fact, fructose and mannose are mutually altered by regulation with mannose-6-phosphate isomerase [38]. The production of glucose was documented as a result of the combination of trehalose and α-lactose, and its relationship with the levels of relevant enzymes and increased amount of glucan endo-1,3-β-D-glucosidase suggested that the buildup of cellobiose favorably occurs during the maturing process of coffee cherries [32]. The aforementioned results suggested that the increase and decrease in sucrose phosphate synthesis in different stages contribute to the flavor of coffee cherries (Figure 3).

**Figure 4:**
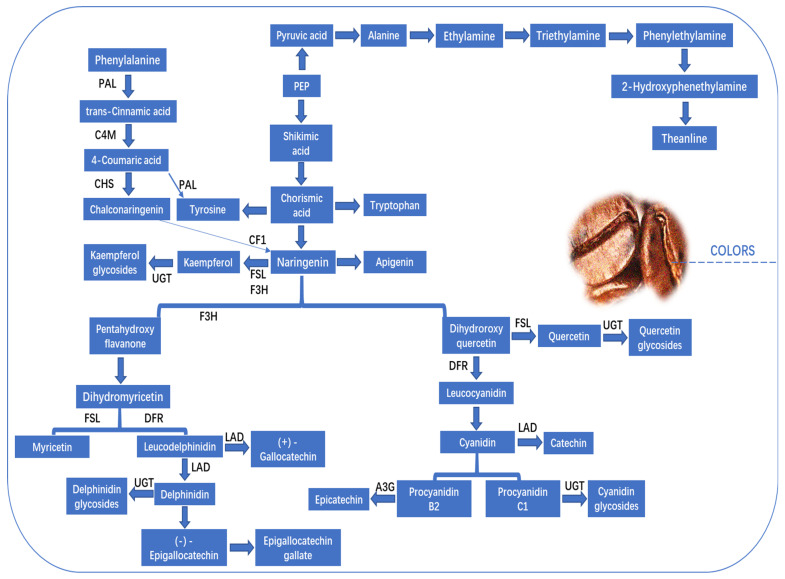
Different compounds that participate in metabolic pathways, contributing to the color of coffee. This figure was adopted from [32] with some modifications.

Flavonoids are a class of polyphenolic secondary metabolites found in plants, including in coffee beans. Flavonoids, and flavanols in particular, have a significant role in the creation of taste, color, and aroma in fruits, particularly in the astringent flavor. Coffee is one of the best sources of other bioactive compounds, such as flavonoids and phenolic acids [39]. The phenylpropanoid and flavonoid biosynthesis pathways are primarily utilized for the purpose of flavonoid production [40]. In arabica coffee cherries, the key proteins and their enzymes are trans-cinnamate 4- monooxygenase, flavonol synthase-like, flavanol 3-O-glucosyltransferase, and chalcone-flavanone isomerase family protein. Phenylalanine ammonia-lyase and phenylalanine increased, while transcinnamic acid decreased. This work suggests that trans-cinnamic acid is the chemical substrate for chalcone and naringenin production. Trans-cinnamate 4-monooxygenase, chalcone synthase, and chalcone-flavanone isomerase family protein bind in the phenylpropanoid pathway, as illustrated in Figure 4. Furthermore, glycosidic chemicals have a crucial role in green coffee beans as they facilitate the release of aglycones during the roasting process, hence enhancing the fragrance profile [41]. The presence of cyanidin glycosides (*n* = 5), delphinidin glucosides (*n* = 2), kaempferol glycosides (*n* = 5), and quercetin glycosides (*n* = 2) was also identified. The glycosides displayed diverse expression patterns throughout the ripening process, with quercetin 3-O-glucoside demonstrating the most pronounced ionic peak intensity across all four stages of coffee. The metabolism of sugar, amino acids, and organic acids in fresh coffee cherries may play a role in the development of floral, fruity, and caramelized aromas, as well as the sweet taste, which are considered potential flavor characteristics. The flavonoid fraction has the potential to significantly enhance the astringent taste and skin color. Furthermore, the environmental conditions, including height, soil composition, duration of daylight, and amount of precipitation, can potentially influence the maturation process of coffee cherries.

## 5. Proteomics Linked to Altitude and Different Environmental Adaptations of Arabica Coffee

The impact of environmental conditions on flavor is undeniably significant. Additionally, the proteomic variations among cultivars, which arise from extended adaptation to specific climatic niches, are expected to have a significant influence on various metabolic processes that contribute to specific aspects of coffee plant and bean quality. There is a consensus among scholars that coffee derived from plants cultivated at elevated altitudes exhibits enhanced flavor characteristics. Recently, in a study published in *Eureka*, the authors utilized the significant advancements in MS/MS technology, computational capabilities, and the availability of proteomic databases that have transpired in recent years to conduct a thorough analysis of the proteomes of *C. arabica* beans obtained from two unique cultivars. The Rwanda Shyira (RS) cultivar was cultivated at high elevations in the Nyabihu district of Western Rwanda, while the Brazil Flor de Ipe (BFDI) cultivar was cultivated at relatively low elevations in the Minas Gerais province of Southeastern Brazil. The researchers documented the initial occurrence of a proteomic disparity that may be associated with the enhanced flavor of high-altitude *C. arabica* [18]. Furthermore, they identified several significant variations that suggest the RS cultivar of *C. arabica* has adapted to both abiotic and biotic stressors caused by high elevation, such as hypoxic, cold, and disease stresses [20]. They found that the disease resistance protein containing an NB-ARC domain exhibited the most significant disparity in expressional intensity, and this homolog was found by conducting a BLAST search with the NB-ARC domain-containing protein (Cc00t22090.1), which was discovered through the coffee genome hub, against the *A. thaliana* proteome. It was chosen since it had the largest query coverage (74%) and the same molecular function as the other homolog. There are three important domains that make up NB-ARC, which is found in the cytosol and the plasma membrane. These domains include a nucleotide-binding (NB) domain, as well as two ARC domains, ARC-1 and ARC-2, which are highly conserved over an extensive range of plant species [42]. A notable disparity in expressional intensity was noted for pyruvate kinase 1 (AT3G52990), a cytosolic enzyme. Notably, an analysis revealed that this protein exhibited more expression in BFDI compared to RS. The cytosolic pyruvate kinase (cPK) is a critical glycolytic enzyme that catalyzes the transfer of phosphate from phosphoenolpyruvate (PEP) to ADP for the synthesis of ATP during glycolysis [43]. A marginal yet noteworthy disparity in the level of expression was also noted for fibrillarin 1 (AT5G52990), which exhibited more expression in RS compared to BFDI. Fibrillarin is an extensively preserved methyltransferase that plays a crucial role in the treatment of primary ribosomal transcripts and is essential for the formation of ribosomes, Additionally, it plays a role in the preliminary stages of the start of ribosomal transcription [44].

Some key proteins such as Glutathione-s-transferases GST, NB-ARC domain-containing protein, fibrillarin, and NADP-Binding Rossmann-fold superfamily protein were also found to be differentially regulated in both *Coffea arabica* cultivars. Moreover, there is evidence to suggest that individuals who consume diets high in coffee, which is abundant in flavonoids, and broccoli, which contains isothiocyanates, see an increase in both the concentration and activity of GST in their saliva. GSTs are likely involved in the detoxification of several compounds present in human saliva [45]. The detoxification of isothiocyanates in Drosophila melanogaster involves the participation of many GSTs. Likewise, the presence of these GSTs may be observed in the gustatory receptors of Rattus norvegicus [20]. In Type II cells, GSTs are particularly localized and are renowned for their role in the detection of bitter, umami, and sweet compounds. This study provides additional support for the notion that GSTs play a significant role in the detection and detoxification of bitter chemicals across many species [46,47]. The quality of coffee is generally linked to more intense non-bitter flavors and a decrease in the overall bitter taste. For example, *C. canephora* beans are more bitter and hence regarded to be of lower quality. Schwartz et al. (2023) reported the capacity of GSTs to facilitate the transfer of glutathione to isothiocyanates in ex vivo human saliva samples. However, it is yet to be determined whether this same mechanism applies to flavonoids [47]. The impact of glutathione transfer to these substrates on the perception of bitterness is currently not fully understood. However, it is probable that this detoxification mechanism also hinders the binding of bitter molecules to their receptors on gustatory cells, similar to the mechanism observed in PRPs. Consequently, it is reasonable to hypothesize that an increase in GST activity in saliva could potentially reduce the sensation of bitterness. The increased expression of the GST domain-containing EF1 in the RS cultivar of *C. arabica* may be linked to the alteration of naturally occurring flavonoids and other bitter compounds, potentially leading to a decrease in the bitterness of coffee made from beans from that cultivar. Moreover, the protein in A. thaliana, which contains the NB-ARC domain, serves as a regulator of immunity and disease resistance by controlling the activity of resistance (R) proteins [48]. This is a critical regulator of R proteins in Nicotiana benthamiana, which activate effector-triggered immunological (ETI) responses to invading pathogens that express avirulent (Avr) genes. The relationship between elevation and plant defenses is a subject of ongoing debate. Some studies have pragmatic constructive links between elevation and plant resistance, whereas others have conveyed conflicting results [49,50]. These varying results are likely attributable to other abiotic factors that are reliant on elevation, such as temperature, rather than altitude itself; for example, the NB-ARC domain-containing disease resistance protein is expressed more in beans of the RS cultivar of *C. arabica* due to the lower temperature range in the Shiyra region of Rwanda, where R protein expression and ETI signaling, which are controlled by the NB-ARC proteins, are commonly observed [20]. According to Bäckström et al. (2007), plant fibrillarin has been demonstrated to function as a component (specifically, subunit 36a) of a multi-protein complex known as the mediator of RNA polymerase II transcription. This complex serves as a channel for communication between transcription control proteins and core promoters [51]. It is worth noting that the mediator16 subunit of the transcriptional coactivator complex has been demonstrated to also play a role in the regulation of gene expression related to cold response in *A. thaliana* [52]. The Shyira region of the Nyabihu district in Rwanda experiences a daytime temperature range of 15–18 °C, which is notably lower compared to the Sul de Minas region in Brazil, owing to its elevated height. The observed increase in fibrillarin expression in the RS cultivar compared to BFDI can perhaps be attributed to the need for enhanced fibrillarin expression in the RS cultivar of C. arabica. This phenomenon may be necessary to effectively regulate Cold On-Regulated (COR) genes in response to lower temperatures [52]. Collectively, the aforementioned literature and data may position fibrillarin as a focal point in an intriguing evolutionary trade-off, wherein the RS cultivar of *C. arabica* exhibits an upregulation of fibrillarin expression in reaction to temperature-induced stressors. However, this upregulation also renders the cultivar more vulnerable to systemic infection caused by umbra viruses, such as the groundnut rosette virus.

## 6. Proteomics Linked to Antioxidative and Drought Responses in Arabica Coffee

Gaining insight into the function of proteins in plants’ reaction to escalating drought is essential for a more comprehensive understanding of acclimation processes and for aiding in breeding initiatives aimed at creating resistant cultivars [53]. Next-generation proteomics has made significant progress in providing a rapid and precise molecular methodology for protein identification and the elucidation of pathways linked to the physiological reactions of biological systems under abiotic stressful circumstances. Prolonged periods of severe drought can result in the demise of plants, while even moderate droughts can exert adverse consequences that affect the cultivation of coffee [54]. A shotgun proteomic analysis was conducted on three wheat cultivars, *Triticum aestivum*, which exhibited variations in their capacity to sustain grain yield under conditions of limited water availability. This analysis unveiled noteworthy disparities in their reaction to drought, which were attributed to alterations in the proteins associated with photosynthesis and the metabolism of oxidative stress [55]. The antioxidant activity of coffee is mainly due to the presence of polyphenols, flavonoids, and other bioactive substances [56]. Examples include caffeine, gallic acid, and proanthocyanidins, which contribute to antioxidant efficacies [57]. Recently, in a study published by Isabel Marques et al. (2022), the authors utilized a label-free proteomic methodology on two farmed genotypes of the predominant coffee species *C. canephora* cv. Conilon clone 153 (CL153) and *C. arabica* L. cv. Icatu Vermelho (Icatu), which exhibit responses to mild and severe water deficiencies [33]. The authors found that proteins like Pathogenesis-related protein (PRP)1B in CL153 and Polyphenol oxidase I and carotenoid 9–10-cleavage dioxygenase in Icatu are typically implicated in response to abiotic stressors and are the most abundant proteins detected under MWD. Plant reactive proteins (PRPs) are essential for defense mechanisms against both biotic and abiotic stimuli; for example, PRPs help rice plants withstand drought and salt, and similarly, polyphenol oxidase (PPO) regulates the redox state of phenolic molecules when plants are stressed, which is an important part of how plants respond to environmental challenges. Isabel Marques et al. (2022) also reported that the more severe water shortage (SWD) led to an increase in the number of differentially abundant proteins (DAPs) in both genotypes. In the context of SWD, the protein that was found in the highest quantity was the NADP-dependent D-sorbitol-6-phosphate dehydrogenase. It was 2.9 times more abundant in CL153 and 1.5 times more numerous in Icatu. Additionally, this protein was the only one that was expressed by both genotypes [33]; this protein is linked to the ability to withstand abiotic stimuli, such as drought and salt [58], and controls the levels of polyols, which are small-chain carbohydrates that function as osmolytes during periods of drought and throughout the recovery process afterward [59]. Additionally, this protein may have a significant impact on the ability of coffee plants to tolerate abiotic stress by controlling the transport and metabolism of polyols [33].

PsbP domain-containing protein 4, chlorophyll a–b binding protein 4, and RuBisCO activase 1 in Icatu also cope with drought stress. The upregulation of RuBisCO activase, a catalytic chaperone responsible for regulating RuBisCO activity, could potentially play a crucial role in the plant’s reaction to modified environmental circumstances. CL153 exhibited a high presence of proteins associated with overall cellular stress responses, such as thioredoxin H-type 1 and xylose isomerase. On the other hand, Icatu increased the expression of proteins involved in abiotic stress responses, such as Serpin-ZX, as well as detoxification processes, such as L-ascorbate peroxidase T and Malate dehydrogenase. Spinins have a significant function in the context of abiotic stress, as evidenced by their interactions with proteases that exhibit responsiveness to desiccation [60]. ROS-detoxifying enzymes are also crucial in situations of water scarcity. When plants undergo drought stress, their cells are subjected to elevated levels of reactive oxygen species (ROS). Cells often employ defensive mechanisms to mitigate the adverse effects of reactive oxygen species (ROS) by minimizing or preventing oxidative damage, hence enhancing their ability to withstand dry conditions. A range of methods can be employed to mitigate the excessive generation of reactive oxygen species (ROS), including enzymatic antioxidant mechanisms such as catalase (CAT), superoxide dismutase (SOD), Ascorbate Peroxidase (APX), peroxidase (POD), and PPO. These processes have the potential to reduce cellular harm. For example, when subjected to both drought and cold stress, the enzymatic activity of CAT, SOD, APX, and glutathione reductase (GR) was found to be increased in many *C. arabica* genotypes, including the Icatu variety [61].

## 7. Proteomics Linked to Somatic Embryogenesis of Arabica Coffee

**Figure 5:**
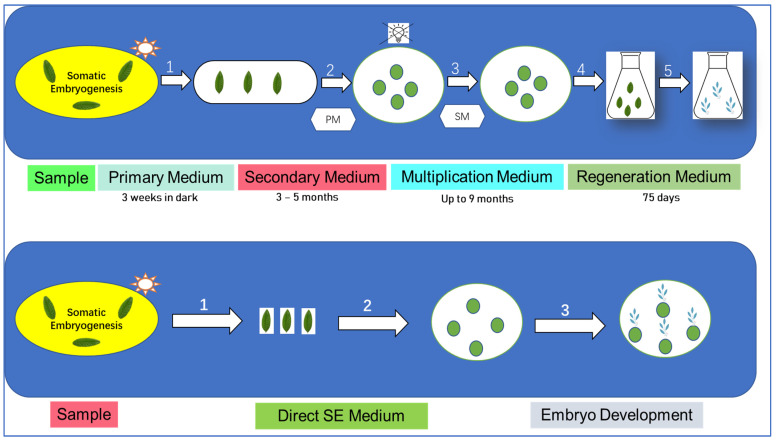
Coffee somatic embryogenesis overview. Above is High-frequency indirect method; Below is low-frequency direct method. This figure was adopted from [65] with some modifications.

The initiation of somatic embryogenesis in all species often occurs through the exposure of plant tissues to the appropriate stimuli, typically in the form of plant hormones. The reprogramming of a differentiated somatic cell can be induced by a proper equilibrium between external hormones and internal factors. However, this equilibrium may also facilitate the proliferation of totipotent undifferentiated cells, which are dormant and found in certain tissues, such as plant stem cells. The conventional idea about the development of somatic embryogenesis posits that somatic cells that have undergone differentiation can retain their ability to produce embryos and be reprogrammed to differentiate into new viable embryos [62]. The scientific investigation of somatic embryogenesis technology in coffee dates back to 1970 [63]. The methods used to induce somatic embryogenesis and promote the growth of embryos are influenced by the genotype, resulting in the creation of specialized protocols for each species by empirical means [64]. According to Campos et al. (2016), a total of 1052 non-redundant proteins were discovered, with 5 of them being designated to possess embryogenic capability [35]. Based on our current understanding, a significant number of proteins lack annotation and remain uncharacterized, indicating a limited understanding of somatic embryogenesis in coffee at the molecular level. This analysis provides valuable insights into the proteome of somatic calli in coffee, serving as an initial step towards comprehending the embryogenic process in coffee as demonstrated in Figure 5.

The annotation of proteins within this review can provide guidance for future investigations in this field. An investigation on the distinctions between embryogenic and non-embryogenic calli is being carried out. There are certain proteins that are only found in embryogenic cell lines and not in non-embryogenic cell lines. These proteins have been proposed as molecular markers for distinct phases of embryogenesis, such as enolase and globulin S11 for the torpedo stage of embryo development. In addition, proteins that are associated with the stress response were discovered. These proteins include HEAT SHOCK PROTEIN (HSP 70) and cytoplasmic aldolase, both of which were found to be more prevalent in the cotyledonary stage. It is well established that stress is a significant element in the process of inducing somatic embryogenesis, and the presence of these proteins provides further evidence [66]. Campos et al. (2017) stated the information to guide the protocols that are currently in use. In order to accomplish this, it is essential to have the appropriate experimental setup, in which the comparison is carried out prior to the cells differentiating into various tissues. An example of a possible experimental setup would be to take a sample from a portion of the cloned calli in which the cytological properties are either similar or, at the very least, not detectable. Through the use of the other component, the differentiation process is finished, and the embryogenic potential of each sample batch is evaluated. Integrated omics analysis needs to be performed on the batches that are intriguing, and correlations can be discovered between the observed embryogenic capability and the transcripts, proteins, or metabolites that are present in the samples. In spite of the fact that the expression of a number of genes has been demonstrated in coffee embryogenic cells, the precise function and regulation of a significant number of these genes are yet unknown [65]. *C. arabica* is traditionally propagated through the use of seeds. Typically, following a breeding procedure lasting a minimum of 20 years, the seed lines are deemed pure and are subsequently marketed in their pure form. The seeds of coffee are classified as non-orthodox, indicating their ability to undergo partial dehydration. However, they are not suitable for long-term storage in traditional gene banks at temperatures below 20 °C. Another excellent substitute is the cryopreservation of seeds, which requires less upkeep than in vitro or field conservation. The utilization of cuttings for propagation is limited to *C. canephora* only. The multiplication rates achieved using cuttings in Arabica coffee remain poor and are not being utilized in commercial applications. The commercial application of somatic embryogenesis for *C. arabica* has been observed in Central America. Currently, around 7 million plantlets originating from somatic embryogenesis are cultivated in Central American fields [67]. However, we did not find any data regarding somatic embryogenesis of coffee in Yunnan province of China, which is also one of the lacking aspects related to coffee cultivation in southwest China. Therefore, it is important that researchers work on coffee areas.

## 8. Lack of Proteomic Studies and Future Direction Associated with Coffee

In comparison to previous proteomics studies linked to horticulture crops, the aforementioned research on coffee is rather recent. Less research has been conducted in the past ten years on proteome profiling in relation to coffee; in reality, most of these studies have only involved one-time coffee samples. Regretfully, no historical research on contemporary technologies and multiple approaches was discovered, including morphological, physiological, biochemical, transcriptomic, and proteomics analyses on coffee plants or seeds at various altitudes, times, and stages of maturity; comparative studies on Arabica vs. Canephor varieties; normal coffee vs. pea berry coffee; biotic and abiotic stresses; and smart agriculture approaches, which made it exceedingly challenging to comprehend genes, proteins, and the correlation between genes and proteins in both normal and fluctuating environments. It is also acknowledged that the vulnerable changes in the coffee belt and other environmental factors could impede the real situation. Variations in the climate may have an impact on the physiological, biochemical, and molecular processes related to coffee. As of right now, no research has been conducted on how coffee plant growth and quality can be affected by reducing abiotic stress. Therefore, these could also be the subject of future research. There is no denying the benefits of using genomics technology in coffee growing, especially in terms of avoiding the laborious and slow process of conventional breeding in favor of sustainable agriculture. In fact, the application of proteomics technology to improve coffee quality, breeding, and growth will boost the coffee beverage industry.

## 9. Conclusions

Coffee, being a highly profitable crop in the beverage market, demonstrates significant promise in the food sector. This review focuses on the application of proteomics in studying coffee and its associated qualities like growth, somatic embryogenesis, environmental adaptation, altitude, drought, and the role of enzymes and protein processes in flavor development. In future studies, researchers should prioritize the examination of coffee’s morphological, physiological, biochemical, metabolomic, transcriptomic, and proteomic components in relation to various features. Conducting a comparative study on various coffee cultivars is crucial in order to analyze their performance under different environmental conditions, altitudes, and geographical locations and at different stages of coffee production (from fresh cherries to green beans to roasted beans). Additionally, it is essential to assess important quality indicators to improve germplasm and breeding technology for both academic research and the food industry.

## Figures and Tables

**Table 1:** The composition of amino acids and proteins associated with coffee.

Serial Number	Amino Acids and Proteins in Coffee	References
	Amino Acids in Coffee	
1	Arginine	
2	Leucine	
3	Phenylalanine	
4	Threonine	
5	Methionine	
6	Lysine	
7	Histidine	
8	Isoleucine	
9	Aspartic acid	[29,30,31,32]
10	Valine	
11	Alanine	
12	Glycine	
13	Proline	
14	Glutamic acid	
15	Tyrosine	
16	Serine	
	Proteins in Coffee	
17	NB-ARC domain-containing protein	
18	Elongation factor1-gamma 2	
19	Mediator of RNA polymerase II transcription subunit 36a-like	
20	Pyruvate kinase 1, cytosolic	
21	Epimerase domain-containing protein	
22	Auxin-binding protein ABP20	
23	Oxygen-evolving enhancer protein 1	
24	Photosystem I reaction center subunit I	[14,27,28,33,34,35]
25	Oxygen-evolving enhancer protein 2	
26	Peptidyl-prolyl cis–trans isomerase	
27	Quinone oxidoreductase-like protein At1g23740	
28	Uncharacterized protein At2g37660	
29	Chlorophyll a–b binding protein CP26	
30	Polyphenol oxidase I	
31	Phosphoglycerate kinase	
32	Glyceraldehyde-3-phosphate dehydrogenase	

## Data Availability

The original contributions presented in the study are included in the article, further inquiries can be directed to the corresponding author.

## References

[1] Chen S., Xiao Y., Tang W., Jiang F., Zhu J., Zhou Y., Ye L. (2023). Evaluation of Physicochemical Characteristics and Sensory Properties of Cold Brew Coffees Prepared Using Ultrahigh Pressure under Different Extraction Conditions. Foods.

[2] Batista M.J.P.A., Marques M.B.F., Franca A.S., Oliveira L.S. (2023). Development of Films from Spent Coffee Grounds’ Polysaccharides Crosslinked with Calcium Ions and 1,4-Phenylenediboronic Acid: A Comparative Analysis of Film Properties and Biodegradability. Foods.

[3] Rocha R.A.R., da Cruz M.A.D., Silva L.C.F., Costa G.X.R., Amaral L.R., Bertarini P.L.L., Gomes M.S., Santos L.D. (2024). Evaluation of Arabica Coffee Fermentation Using Machine Learning. Foods.

[4] Poláková K., Bobková A., Demianová A., Bobko M., Lidiková J., Jurčaga L., Belej Ľ., Mesárošová A., Korčok M., Tóth T. (2023). Quality Attributes and Sensory Acceptance of Different Botanical Coffee Co-Products. Foods.

[5] van der Vossen H., Bertrand B., Charrier A. (2015). Next Generation Variety Development for Sustainable Production of Arabica Coffee (*Coffea arabica* L.): A Revie. Euphytica.

[6] Silva L.C.F., Pereira P.V.R., da Cruz M.A.D., Costa G.X.R., Rocha R.A.R., Bertarini P.L.L., do Amaral L.R., Gomes M.S., Santos L.D. (2024). Enhancing Sensory Quality of Coffee: The Impact of Fermentation Techniques on *Coffea arabica* Cv. Catiguá MG2. Foods.

[7] Coelho E.G., Bertarini P.L.L., Gomes M.S., Amaral L.R., Zotarelli M.F., Santos L.D., Santana R.C. (2024). Physicochemical and Sensory Properties of *Arabica* Coffee Beans of Arara Cv. Dried Using Different Methods. Foods.

[8] Schmidt R., da Silva C.A., Silva L.O.E., Espindula M.C., Rodrigues W.P., Vieira H.D., Tomaz M.A., Partelli F.L. (2023). Accumulation of Nutrients and the Relation between Fruit, Grain, and Husk of Coffee Robusta Cultivated in Brazilian Amazon. Plants.

[9] Min C., Biyi M., Jianneng L., Yimin L., Yijun L., Long C. (2022). Characterization of the Volatile Organic Compounds Produced from Green Coffee in Different Years by Gas Chromatography Ion Mobility Spectrometry. RSC Adv..

[10] Zhai H., Dong W., Tang Y., Hu R., Yu X., Chen X. (2024). Characterization of the Volatile Flavour Compounds in Yunnan Arabica Coffee Prepared by Different Primary Processing Methods Using HS-SPME/GC-MS and HS-GC-IMS. LWT.

[11] Caixeta E. (2020). Selective Efficiency of Genome-Wide Selection in *Coffea canephora* Breeding. Tree Genet. Genomes.

[12] Huded A.K.C., Jingade P., Bychappa M., Mishra M.K. (2020). Genetic Diversity and Population Structure Analysis of Coffee (*Coffea canephora*) Germplasm Collections in Indian Gene Bank Employing SRAP and SCoT Markers. Int. J. Fruit Sci..

[13] Berni R., Charton S., Planchon S., Legay S., Romi M., Cantini C., Cai G., Hausman J.-F., Renaut J., Guerriero G. (2021). Molecular Investigation of Tuscan Sweet Cherries Sampled over Three Years: Gene Expression Analysis Coupled to Metabolomics and Proteomics. Hortic. Res..

[14] Montavon P., Mauron A.-F., Duruz E. (2003). Changes in Green Coffee Protein Profiles during Roasting. J Agric Food Chem.

[15] Anthony F., Clifford M.N., Noirot M. (1993). Biochemical Diversity in the Genus *Coffea* L.: Chlorogenic Acids, Caffeine and Mozambioside Contents. Genet. Resour. Crop Evol..

[16] Coffee: Botany, Biochemistry and Production of Beans and Beverage [1 ed.] 9781461566571, 1461566576. https://ebin.pub/coffee-botany-biochemistry-and-production-of-beans-and-beverage-1nbsped-9781461566571-1461566576.html.

[17] Mazzafera P., Schimpl F., Kiyota E. (2019). Proteins of Coffee Beans: Recent Advances.

[18] Livramento K., Borém F., Torres L., Silva F., Livramento D., Paiva L. (2017). Proteomic Analysis of Natural and Demucilaged Coffee Beans from Plantations at Different Altitudes in the Mantiqueira Mountains. J. Exp. Agric. Int..

[19] Olechno E., Puścion-Jakubik A., Zujko M.E., Socha K. (2021). Influence of Various Factors on Caffeine Content in Coffee Brews. Foods.

[20] Fenrich C., Lauman P., Wickramasinghe P. (2023). Proteomic Analysis of Higher & Lower Altitude Cultivars of *Coffea arabica* Reveals Differences Related to Environmental Adaptations and Coffee Bean Flavour. Eureka.

[21] Hendre P.S., Aggarwal R.K. (2014). Development of Genic and Genomic SSR Markers of Robusta Coffee (*Coffea canephora* Pierre Ex A. Froehner). PLoS ONE.

[22] ME THAI|Bangkok. https://methaicoffee.com/lander.

[23] Martínez-Esteso M.J., Martínez-Márquez A., Sellés-Marchart S., Morante-Carriel J.A., Bru-Martínez R. (2015). The Role of Proteomics in Progressing Insights into Plant Secondary Metabolism. Front. Plant Sci..

[24] Mathivanan S. (2021). Abiotic Stress-Induced Molecular and Physiological Changes and Adaptive Mechanisms in Plants. Abiotic Stress in Plants.

[25] Yadav B.G., Aakanksha, Kumar R., Yadava S.K., Kumar A., Ramchiary N. (2023). Understanding the Proteomes of Plant Development and Stress Responses in Brassica Crops. J. Proteome Res..

[26] Vanderschuren H., Lentz E., Zainuddin I., Gruissem W. (2013). Proteomics of Model and Crop Plant Species: Status, Current Limitations and Strategic Advances for Crop Improvement. J. Proteom..

[27] Montavon P., Duruz E., Rumo G., Pratz G. (2003). Evolution of Green Coffee Protein Profiles with Maturation and Relationship to Coffee Cup Quality. J. Agric. Food Chem..

[28] Kerler J., Winkel C., Davidek T., Blank I., Taylor A.J., Linforth R.S.T. (2010). Basic Chemistry and Process Conditions for Reaction Flavours with Particular Focus on Maillard-Type Reactions. Food Flavour Technology.

[29] Dong W., Tan L., Zhao J., Hu R., Lu M. (2015). Characterization of Fatty Acid, Amino Acid and Volatile Compound Compositions and Bioactive Components of Seven Coffee (Coffea Robusta) Cultivars Grown in Hainan Province, China. Molecules.

[30] Bhattarai R.R., Al-Ali H., Johnson S.K. (2022). Extraction, Isolation and Nutritional Quality of Coffee Protein. Foods.

[31] Fitri, Laga A., Dwyana Z., Tawali A.B. (2021). Composition of Amino Acids and Fatty Acids on Luwak Coffee Processing. Food Res..

[32] Li Z., Zhou B., Zheng T., Zhao C., Shen X., Wang X., Qiu M., Fan J. (2023). Integrating Metabolomics and Proteomics Technologies Provides Insights into the Flavor Precursor Changes at Different Maturity Stages of Arabica Coffee Cherries. Foods.

[33] Marques I., Gouveia D., Gaillard J.-C., Martins S., Semedo M.C., Lidon F.C., DaMatta F.M., Ribeiro-Barros A.I., Armengaud J., Ramalho J.C. (2022). Next-Generation Proteomics Reveals a Greater Antioxidative Response to Drought in *Coffea arabica* Than in *Coffea canephora*. Agronomy.

[34] Takahashi S., Saito K., Li X., Jia H., Kato H. (2022). iTRAQ-Based Quantitative Proteomics Reveals the Energy Metabolism Alterations Induced by Chlorogenic Acid in HepG2 Cells. Nutrients.

[35] Campos N.A., Paiva L.V., Panis B., Carpentier S.C. (2016). The Proteome Profile of Embryogenic Cell Suspensions of *Coffea arabica* L.. Proteomics.

[36] Wang Y., Wang X., Hu G., Hong D., Bai X., Guo T., Zhou H., Li J., Qiu M. (2021). Chemical Ingredients Characterization Basing on 1H NMR and SHS-GC/MS in Twelve Cultivars of *Coffea arabica* Roasted Beans. Food Res. Int..

[37] Chen D., Sun Z., Gao J., Peng J., Wang Z., Zhao Y., Lin Z., Dai W. (2022). Metabolomics Combined with Proteomics Provides a Novel Interpretation of the Compound Differences among Chinese Tea Cultivars (*Camellia sinensis* var. *sinensis*) with Different Manufacturing Suitabilities. Food Chem.

[38] Tian X., Zhu L., Yang N., Song J., Zhao H., Zhang J., Ma F., Li M. (2021). Proteomics and Metabolomics Reveal the Regulatory Pathways of Ripening and Quality in Post-Harvest Kiwifruits. J. Agric. Food Chem..

[39] Górecki M., Hallmann E. (2020). The Antioxidant Content of Coffee and Its In Vitro Activity as an Effect of Its Production Method and Roasting and Brewing Time. Antioxidants.

[40] Li X., Jiang J., Chen Z., Jackson A. (2021). Transcriptomic, Proteomic and Metabolomic Analysis of Flavonoid Biosynthesis during Fruit Maturation in Rubus Chingii Hu. Front. Plant Sci..

[41] Haure M., Nguyen T.K.C., Cendres A., Perino S., Licandro H., Waché Y. (2022). Glycosidically Bound Volatile Profiles of Green and Roasted Coffee Beans and Aromatic Potential of the Spent Coffee Ground. Eur. Food Res. Technol..

[42] Subcellular Localisation Database for Arabidopsis Proteins Version 5. https://research-repository.uwa.edu.au/en/datasets/subcellular-localisation-database-for-arabidopsis-proteins-versio-3.

[43] Wulfert S., Schilasky S., Krueger S. (2020). Transcriptional and Biochemical Characterization of Cytosolic Pyruvate Kinases in *Arabidopsis thaliana*. Plants.

[44] Yildirim S., Castano E., Sobol M., Philimonenko V.V., Dzijak R., Venit T., Hozák P. (2013). Involvement of Phosphatidylinositol 4,5-Bisphosphate in RNA Polymerase I Transcription. J. Cell Sci..

[45] Sreerama L., Hedge M.W., Sladek N.E. (1995). Identification of a Class 3 Aldehyde Dehydrogenase in Human Saliva and Increased Levels of This Enzyme, Glutathione S-Transferases, and DT-Diaphorase in the Saliva of Subjects Who Continually Ingest Large Quantities of Coffee or Broccoli. Clin. Cancer Res..

[46] Gonzalez D., Fraichard S., Grassein P., Delarue P., Senet P., Nicolaï A., Chavanne E., Mucher E., Artur Y., Ferveur J.-F. (2018). Characterization of a Drosophila Glutathione Transferase Involved in Isothiocyanate Detoxification. Insect Biochem. Mol. Biol..

[47] Schwartz M., Boichot V., Fraichard S., Muradova M., Senet P., Nicolai A., Lirussi F., Bas M., Canon F., Heydel J.-M. (2023). Role of Insect and Mammal Glutathione Transferases in Chemoperception. Biomolecules.

[48] Cunningham F., Allen J.E., Allen J., Alvarez-Jarreta J., Amode M.R., Armean I.M., Austine-Orimoloye O., Azov A.G., Barnes I., Bennett R. (2022). Ensembl 2022. Nucleic Acids Res..

[49] Wu L., Chen H., Curtis C., Fu Z.Q. (2014). Go in for the Kill: How Plants Deploy Effector-Triggered Immunity to Combat Pathogens. [Corrected]. Virulence.

[50] Cheng C., Gao X., Feng B., Sheen J., Shan L., He P. (2013). Plant Immune Response to Pathogens Differs with Changing Temperatures. Nat. Commun..

[51] Bäckström S., Elfving N., Nilsson R., Wingsle G., Björklund S. (2007). Purification of a Plant Mediator from *Arabidopsis thaliana* Identifies PFT1 as the Med25 Subunit. Mol. Cell.

[52] Hemsley P.A., Hurst C.H., Kaliyadasa E., Lamb R., Knight M.R., De Cothi E.A., Steele J.F., Knight H. (2014). The *Arabidopsis* Mediator Complex Subunits MED16, MED14, and MED2 Regulate Mediator and RNA Polymerase II Recruitment to CBF-Responsive Cold-Regulated Genes. Plant Cell.

[53] Hasan M.M.-U., Ma F., Prodhan Z.H., Li F., Shen H., Chen Y., Wang X. (2018). Molecular and Physio-Biochemical Characterization of Cotton Species for Assessing Drought Stress Tolerance. Int. J. Mol. Sci..

[54] dos Santos C.S., de Freitas A.F., da Silva G.H.B., Pennacchi J.P., Figueiredo de Carvalho M.A., Santos M.d.O., Junqueira de Moraes T.S., de Rezende Abrahão J.C., Pereira A.A., Carvalho G.R. (2023). Phenotypic Plasticity Index as a Strategy for Selecting Water-Stress-Adapted Coffee Genotypes. Plants.

[55] Ford K.L., Cassin A., Bacic A. (2011). Quantitative Proteomic Analysis of Wheat Cultivars with Differing Drought Stress Tolerance. Front. Plant Sci..

[56] Lapčíková B., Lapčík L., Barták P., Valenta T., Dokládalová K. (2023). Effect of Extraction Methods on Aroma Profile, Antioxidant Activity and Sensory Acceptability of Specialty Coffee Brews. Foods.

[57] Yang K., Zhang L., Liao P., Xiao Z., Zhang F., Sindaye D., Xin Z., Tan C., Deng J., Yin Y. (2020). Impact of Gallic Acid on Gut Health: Focus on the Gut Microbiome, Immune Response, and Mechanisms of Action. Front. Immunol..

[58] Jia Y., Wong D.C., Sweetman C., Bruning J.B., Ford C.M. (2015). New Insights into the Evolutionary History of Plant Sorbitol Dehydrogenase. BMC Plant Biol..

[59] Yancey P.H., Clark M.E., Hand S.C., Bowlus R.D., Somero G.N. (1982). Living with Water Stress: Evolution of Osmolyte Systems. Science.

[60] Srinivasan T., Kumar K.R.R., Kirti P.B. (2009). Constitutive Expression of a Trypsin Protease Inhibitor Confers Multiple Stress Tolerance in Transgenic Tobacco. Plant Cell Physiol..

[61] Ramalho J.C., Rodrigues A.P., Lidon F.C., Marques L.M.C., Leitão A.E., Fortunato A.S., Pais I.P., Silva M.J., Scotti-Campos P., Lopes A. (2018). Stress Cross-Response of the Antioxidative System Promoted by Superimposed Drought and Cold Conditions in *Coffea* spp.. PLoS ONE.

[62] Yang X., Zhang X. (2010). Regulation of Somatic Embryogenesis in Higher Plants. Crit. Rev. Plant Sci..

[63] Staritsky G. (1970). EMBRYOID FORMATION IN CALLUS TISSUES OF COFFEE. Acta Bot. Neerl..

[64] Santana-Buzzy N., Rojas-Herrera R., Galaz-Ávalos R.M., Ku-Cauich J.R., Mijangos-Cortés J., Gutiérrez-Pacheco L.C., Canto A., Quiroz-Figueroa F., Loyola-Vargas V.M. (2007). Advances in Coffee Tissue Culture and Its Practical Applications. In Vitro Cell. Dev. Biol.-Plant.

[65] Campos N.A., Panis B., Carpentier S.C. (2017). Somatic Embryogenesis in Coffee: The Evolution of Biotechnology and the Integration of Omics Technologies Offer Great Opportunities. Front. Plant Sci..

[66] Tonietto Â., Sato J.H., Teixeira J.B., De Souza E.M., Pedrosa F.O., Franco O.L., Mehta A. (2012). Proteomic Analysis of Developing Somatic Embryos of *Coffea arabica*. Plant Mol. Biol. Rep..

[67] Etienne H., Bertrand B., Dechamp E., Maurel P., Georget F., Guyot R., Breitler J.C. (2016). Are genetics and epigenetic instabilities of plant embryogenic cells a fatality? The experience of coffee somatic embryogenesis. Hum. Genet. Embryol..

